# Digital Mental Health for Schizophrenia and Other Severe Mental Illnesses: An International Consensus on Current Challenges and Potential Solutions

**DOI:** 10.2196/57155

**Published:** 2024-05-08

**Authors:** Katharine A Smith, Amy Hardy, Anastasia Vinnikova, Charlotte Blease, Lea Milligan, Diego Hidalgo-Mazzei, Sinéad Lambe, Lisa Marzano, Peter J Uhlhaas, Edoardo G Ostinelli, Gerard Anmella, Caroline Zangani, Rosario Aronica, Bridget Dwyer, John Torous, Andrea Cipriani

**Affiliations:** 1 Department of Psychiatry University of Oxford Oxford United Kingdom; 2 Oxford Health NHS Foundation Trust Warneford Hospital Oxford United Kingdom; 3 Oxford Precision Psychiatry Lab NIHR Oxford Health Biomedical Research Centre Oxford United Kingdom; 4 Department of Psychology Institute of Psychiatry, Psychology and Neuroscience King’s College London London United Kingdom; 5 South London & Maudsley NHS Foundation Trust London United Kingdom; 6 MQ Mental Health Research London United Kingdom; 7 Participatory eHealth and Health Data Research Group, Department of Women's and Children's Health Uppsala University Uppsala Sweden; 8 Division of Digital Psychiatry Beth Israel Deaconess Medical Center Harvard Medical School Boston, MA United States; 9 Department of Psychiatry and Psychology Institute of Neuroscience Hospital Clínic de Barcelona Barcelona Spain; 10 Bipolar and Depressive Disorders Unit Digital Innovation Group Institut d’Investigacions Biomèdiques August Pi i Sunyer (IDIBAPS) Barcelona Spain; 11 Centro de Investigación Biomédica en Red de Salud Mental (CIBERSAM) Instituto de Salud Carlos III Madrid Spain; 12 Department of Medicine School of Medicine and Health Sciences Institute of Neurosciences, University of Barcelona Barcelona Spain; 13 Department of Experimental Psychology University of Oxford Oxford United Kingdom; 14 School of Science and Technology Middlesex University London United Kingdom; 15 Institute of Neuroscience and Psychology University of Glasgow Glasgow United Kingdom; 16 Department of Child and Adolescent Psychiatry Charité – Universitätsmedizin Berlin corporate member of Freie Universität Berlin and Humboldt-Universität zu Berlin Berlin Germany; 17 Psychiatry Unit Department of Neurosciences and Mental Health Ospedale Maggiore Policlinico Ca’ Granda, Fondazione Istituto di Ricovero e Cura a Carattere Scientifico Milan Italy; 18 Department of Pathophysiology and Transplantation University of Milan Milan Italy

**Keywords:** digital, mental health, severe mental illness, consensus, lived experience, ethics, user-centered design, patient and public involvement, mobile phone

## Abstract

**Background:**

Digital approaches may be helpful in augmenting care to address unmet mental health needs, particularly for schizophrenia and severe mental illness (SMI).

**Objective:**

An international multidisciplinary group was convened to reach a consensus on the challenges and potential solutions regarding collecting data, delivering treatment, and the ethical challenges in digital mental health approaches for schizophrenia and SMI.

**Methods:**

The consensus development panel method was used, with an in-person meeting of 2 groups: the expert group and the panel. Membership was multidisciplinary including those with lived experience, with equal participation at all stages and coproduction of the consensus outputs and summary. Relevant literature was shared in advance of the meeting, and a systematic search of the recent literature on digital mental health interventions for schizophrenia and psychosis was completed to ensure that the panel was informed before the meeting with the expert group.

**Results:**

Four broad areas of challenge and proposed solutions were identified: (1) user involvement for real coproduction; (2) new approaches to methodology in digital mental health, including agreed standards, data sharing, measuring harms, prevention strategies, and mechanistic research; (3) regulation and funding issues; and (4) implementation in real-world settings (including multidisciplinary collaboration, training, augmenting existing service provision, and social and population-focused approaches). Examples are provided with more detail on human-centered research design, lived experience perspectives, and biomedical ethics in digital mental health approaches for SMI.

**Conclusions:**

The group agreed by consensus on a number of recommendations: (1) a new and improved approach to digital mental health research (with agreed reporting standards, data sharing, and shared protocols), (2) equal emphasis on social and population research as well as biological and psychological approaches, (3) meaningful collaborations across varied disciplines that have previously not worked closely together, (4) increased focus on the business model and product with planning and new funding structures across the whole development pathway, (5) increased focus and reporting on ethical issues and potential harms, and (6) organizational changes to allow for true communication and coproduction with those with lived experience of SMI. This study approach, combining an international expert meeting with patient and public involvement and engagement throughout the process, consensus methodology, discussion, and publication, is a helpful way to identify directions for future research and clinical implementation in rapidly evolving areas and can be combined with measurements of real-world clinical impact over time. Similar initiatives will be helpful in other areas of digital mental health and similarly fast-evolving fields to focus research and organizational change and effect improved real-world clinical implementation.

## Introduction

### Background

Addressing the shortfall in service provision in mental health is a key challenge [1] that has been brought into even sharper focus in the aftermath of the COVID-19 pandemic. The pandemic showed that a shift to digital platforms to deliver synchronous mental health services could be rapidly implemented [2] and can be an acceptable format for many clinicians, patients, and carers [3,4]. However, asynchronous digital approaches (eg, measuring symptoms using digital phenotyping or ecological momentary assessment or providing partially automated therapies using digital platforms) show even more potential to increase capacity and outcomes [5]. These innovations allow patients to undertake a variety of clinically relevant tasks (such as self-monitoring or therapy tasks) outside the in-person clinical encounter. This can be completed independently or with the support of digital navigators or technicians [6] and could reduce the need for specialist clinician support. However, despite their potential, these approaches are mostly still in development and have often proved to be challenging to implement in real-world clinical and community settings [6].

An additional challenge for mental health services is to ensure that the potential benefits of digital approaches are applied in the areas of greatest need, particularly because the pandemic has worsened preexisting disparities in mental health care [7]. Increasing access to care for people with severe mental illnesses (SMIs) such as psychosis and bipolar disorder is a priority worldwide for mental health, particularly in low- and middle-income countries (LMICs) [8]. Although people with SMI experience major health inequalities and have a life expectancy 10 to 25 years shorter than that of the general population [9,10], less research on digital interventions has been conducted in this patient group compared with other conditions [11]. Assumptions that people with SMI will not be able or willing to engage in technology-augmented assessment and treatment have combined with the known element of digital exclusion due to the intersection of SMI and socioeconomic inequities, resulting in a shortfall of development in this area [12,13].

Internet access among people with SMI is increasing [14,15], and ownership of smartphones is now more common than ownership of computers [16]. However, there are still barriers to digital access and digital literacy in this group [17,18]. Even with a device, many people with SMI have insufficient economic resources to maintain consistent access or may lack the confidence or skills to use it to its full ability [12,18,19]. Although there are examples of training programs in digital skills and confidence for people with SMI [20,21] and evidence that they are willing and able to engage effectively with digital mental health [22-25], this is often a neglected area [11].

### Study Objectives

In this study, we used a consensus method to identify the challenges and potential solutions in the development and implementation of digital mental health in the care pathways of people with schizophrenia and other SMIs, and consider how new research could help fill the gap between need and service provision to improve patients’ outcomes. To explore this, we convened an expert international multidisciplinary group to focus specifically on the complexities of collecting data, delivering treatment, and the ethical challenges in this area. We focused on SMI as a group where there is arguably the greatest need. While SMI is a broad term covering mental illnesses causing serious functional impairment, we focused primarily on evidence and examples of digital mental health interventions for schizophrenia and psychosis, and related risks such as suicide.

## Methods

### Overview

We used the consensus development panel (or consensus development conference [CDC]) approach [26,27] and followed the methodology described and used by the US National Institutes of Health [28] and the World Health Organization [26,29]. The CDCs were developed by the National Institutes of Health [26] and we chose this as it is a particularly effective consensus method for identifying broad areas of challenge and potential solutions. This is in contrast to alternative consensus approaches (such as the Delphi or nominal group techniques) that aim to achieve specific criteria or protocols [30]. Therefore, the CDC is a particularly relevant method for a rapidly developing area such as digital mental health [6]. In addition, it enables a multidisciplinary approach and can moderate potential bias from a group of individual experts using several strategies, such as the inclusion of a separate panel of nonexpert participants (hereafter, “the panel”).

Central to the methodology of the CDC is a face-to-face meeting between the expert group and the panel involving an interactive method to develop a consensus. Panel members are provided with evidence by the expert group. The panel members ask questions to clarify and then deliberate on the issue directed by their chairperson in the process to reach a consensus [26].

**Figure 1:**
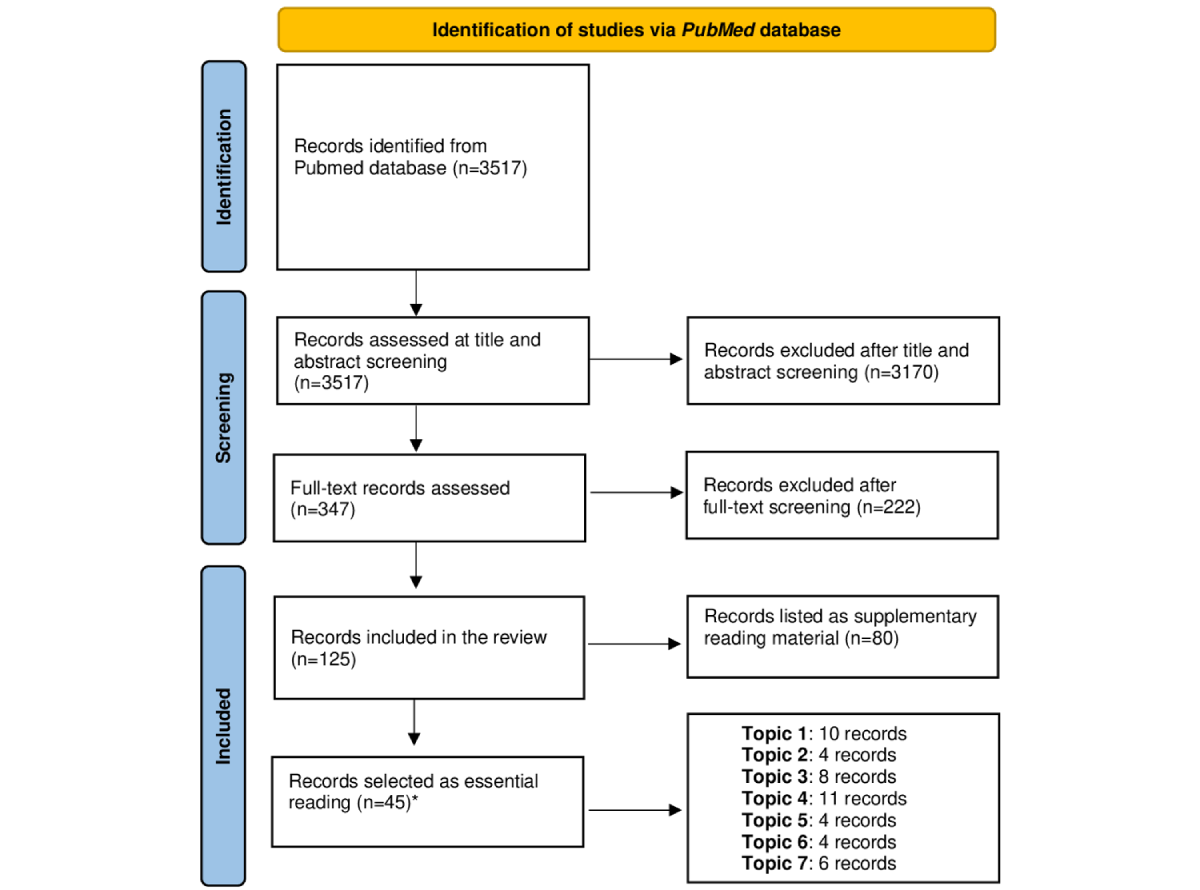
PRISMA (Preferred Reporting Items for Systematic Reviews and Meta-Analyses) flowchart. *Two of the records were each allocated to two topics as they were relevant to both.

In this study, the panel also increased their knowledge of the field in advance of the meeting by conducting a literature review using PubMed to search for terms relevant to the main themes identified by the experts (see Textbox 1 and Figure 1 for details). This preliminary work identified the areas of recent development, uncertainties, or challenges that formed the agenda for the questions to be addressed in the face-to-face meeting.

**Literature review.**

**Methods**

- The panel members conducted a literature review of the records published over the last 5 years (from January 1, 2018) on PubMed, and searched for articles relevant to the themes identified by the expert group in the area of digital technologies and severe mental illness (SMI; focusing for this review on schizophrenia and psychosis).
- The seven identified topics suggested by the experts were (1) digital markers and personalized interventions, (2) patient and public involvement and engagement perspectives on SMI, (3) digital technologies and SMI, (4) virtual reality approaches for SMI, (5) technology and mobile health for suicide prevention, (6) digital approaches to empathy in clinician-patient interactions, and (7) the use of web-based screening to detect emerging psychosis.
- The panel used a broad search strategy for papers relevant to schizophrenia, psychosis, and digital mental health. The search was conducted on September 17, 2023, using the following search strategy: (“Schizophrenia Spectrum and Other Psychotic Disorders” [MeSH Terms] OR (“schizophreni*”[Title/Abstract] OR “psychos*”[Title/Abstract] OR “psychotic”[Title/Abstract])) AND (“digital*”[Title/Abstract] OR “smartphone*”[Title/Abstract] OR “mobile*”[Title/Abstract] OR “virtual*”[Title/Abstract] OR “internet*”[Title/Abstract]) AND 2018/01/01:3000/12/31[Date – Publication].
- The screening process was completed independently by the 7 panelists and any queries were resolved through team discussion. Papers were eligible for inclusion if they were relevant to schizophrenia or psychosis and digital mental health. The search resulted in 3517 records, 3170 (90.13%) of which were excluded after title and abstract screening.
- At the full-text screening, 125 records met the inclusion criteria. Of these 125 records, 45 (36%) were further selected as essential reading by the panel and shared with all the panelists, including a selection of up to 5 articles suggested by each member of the expert group. The remaining 64% (80/125) of the records were listed as supplementary reading material for consultation purposes (see the PRISMA [Preferred Reporting Items for Systematic Reviews and Meta-Analyses] flow diagram [31] in Figure 1).

### The Consensus Meeting

The meeting was held in Rome over 2 days in October 2023. The meeting was facilitated by JT and AC who led the expert group, and by the chair of the panel (KAS) who also recorded and summarized the meeting. Each expert gave a brief presentation, including shared slides, methodology, analysis of data, and relevant citations, followed by a whole-group discussion led by a different member of the panel for each talk. At the end of each day a summary was prepared in discussion with the whole group, and the structure of the consensus (challenges and potential solutions) was agreed upon at the end of the second day. Consensus was defined as either fully met or not met at all, with the outcome being transparently reported [32,33]. At the end of the meeting, the whole group engaged in plenary discussion to identify the key themes and structure the results.

### The Expert Group

The 9 experts (AC, AH, CB, DH, SL, L Milligan, L Marzano, JT, and PU) encompassed expertise in a variety of specialist areas within SMI and digital mental health (including virtual reality, coproduction and co-design, suicide prevention, web-based screening and early intervention, digital approaches to empathy and the therapeutic relationship, ethical issues, and lived experience). The group composition was gender-balanced, and professional backgrounds included psychiatry, psychology, methodology, evidence synthesis, patient and public involvement (PPI), and ethics. The expert group was international (including members from Germany, Italy, Ireland, Spain, Sweden, the United Kingdom, and the United States). In advance of the meeting the experts were asked to provide information on their area of expertise as a short abstract supported by up to 5 references that they considered to be key in the last 2 to 3 years, and this was circulated to the expert group and panel. This was supplemented by a systematic search of the literature conducted by the panel (Textbox 1).

### The Panel

The panel was composed of 7 members (GA, RA, BD, EGO, KAS, AV, and CZ), including early-career and more experienced clinicians and researchers (at a different level of expertise) and an individual with lived experience. The panel members were chosen because they were well informed or experienced in the field of SMI but had no particular expertise in any one area of digital psychiatry. The panel was also international (including members from Italy, Paraguay, Spain, the United Kingdom, and the United States).

In preparation for the meeting, the panel members summarized the best available recent evidence on the 7 topics to be discussed. This was achieved by completing a systematic search of the recent literature in the areas to be covered by the meeting (see Textbox 1 and Figure 1 for details). All panelists were asked to read the selected papers, and each panel member led the group discussion on one expert talk to facilitate equal contributions from members of the expert group and the panel.

### Reflexivity Statement

The meeting was convened by JT and AC, who selected the expert group to represent a balance of professional backgrounds, areas of specialist digital mental health expertise, lived experience, and gender. Panel members were suggested by members of the expert group and through professional contacts. JT and AC were assisted by KAS in the organization and preparation of the meeting. The logistics of the meeting were supported externally by Angelini Pharma, but they did not have any input in the design of the meeting, identification or selection of the expert group or panel, agenda of the meeting, discussions, consensus, or output. We acknowledge that the shared knowledge and experiences of the expert group and panel may have had an impact on the interpretation of the data.

### Ethical Considerations

Ethical approval was not sought for this study as it did not involve research on human participants. The consensus was conference based, and all attendees offered contributions to the research topic in an open environment where talks were voluntary. No personal details were solicited or reported, and all presentations were based on expertise, including the lived experience expert coinvestigator who spoke in her capacity as an expert in how patients with SMI navigate health systems.

## Results

### Overview

The group achieved full consensus on all the outputs of the meeting, identified 4 broad themes, and divided each consensus theme into the current challenges and potential solutions, with examples from existing projects for SMI. A summary of the results is presented in Textbox 2.

**Summary of consensus themes, challenges, and potential solutions.**

**Theme 1: user involvement for real coproduction**

**Challenges:**

- User engagement:
  - Most apps are discarded within days
  - There is a lack of consensus or standards for assessing and monitoring engagement and attrition
  - Most of the research and development of digital tools focuses on conditions outside severe mental illness and is conducted and published in higher-income countries (not low- and middle-income countries)
  - Interventions need to be at a younger age and across the life span

**Potential solutions:**

- Patient and public involvement and active engagement in human-centered design:
  - True representation of diverse populations
  - Coproduction
  - Patient and public involvement and engagement which is integral within the study
- Personalized approaches
- Early interventions

**Theme 2: research methodology**

**Challenges:**

- There is a lack of validated and agreed standards for digital biomarkers or behavioral data

**Potential solutions:**

- Agreed standards for digital mental health research
- Transparent data processing pipelines and data sharing
- Measuring harms
- A focus on prevention and longer-term strategies
- Mechanistic research in digital mental health interventions

**Theme 3: regulation, governance, and funding**

**Challenges:**

- Achieving sustainable funding
- Unstable regulation

**Potential solutions:**

- Sustainability planning in funding calls and applications
- Governance change
- Guidelines for best practice and research standards

**Theme 4: implementation in real-world clinical settings**

**Challenges:**

- Translating research findings into the clinical setting

**Potential solutions:**

- Collaboration between multidisciplinary groups
- Training and assessment in clinics

### Theme 1: User Involvement for Real Coproduction

#### Current Challenges

##### User Engagement

User engagement is a key issue as it represents a potential mechanism of change for improving clinical outcomes in digital health interventions [34,35]. Even in the general population, most apps are discarded within days of first use [36], and recently some have labeled engagement as the “Achilles Heel” of digital therapeutics for mental health, with attrition also of concern [37]. An additional complication is that engagement and adherence are often conflated; participants may disengage for multiple reasons (including the attainment of their goals rather than lack of adherence). The concept of optimal use may be a more representative indicator but needs to be defined a priori in research studies [38]. However, there is also a lack of consensus or standards for assessing and monitoring engagement with and attrition from digital tools, limiting progress in the field [39]. In addition, most of the research on and development of digital tools focuses on conditions outside SMI [11]. Digital technologies, particularly smartphone apps, have the potential to be novel tools for managing SMIs, especially in LMICs [40]. However, most research studies in this area are conducted and published in higher-income countries, and samples may not be representative of the wider SMI population [41]. Individual reports from researchers in LMICs suggest that engagement is also challenging in these settings [42].

##### Interventions at a Younger Age and Across the Life Span

Most mental disorders begin during adolescence [43] and are frequently preceded by subthreshold symptoms, suggesting that this period is critical for early intervention and diagnosis [44]. While this age group may be particularly suited to digital interventions (eg, 46% of individuals aged 13-17 years in the United States reported “almost constant” use of social networking services [45]), effective digital interventions may need to be targeted earlier than in previous studies and tailored to the needs of young people [46,47]. Youth is not the only stage of the life span in which focus is needed. In general, older people are most likely to be digitally excluded, and enduring or treatment-resistant psychosis has received less focus, funding, and innovation for digital approaches [18].

#### Potential Solutions

##### PPI and Engagement in Human-Centered Design

A potential solution to the challenges of user engagement with digital tools is to actively involve patients and participants in the design and development of digital tools and all stages of the research process (see Textbox 3 for further information from a lived experience perspective).

Not only does this active engagement and involvement result in research that is more relevant and useful for users, but it also meets the essential rights of users to be included in the development of interventions that will affect their lives and those closest to them [49]. However, even when users have been included, there is often a lack of true representation of diverse populations [50], resulting in digital solutions that do not provide sufficient options for different individual preferences and life circumstances. PPI and engagement (PPIE) within digital development and research studies can be highly variable, and although the aim is coproduction, for true design *with* and not *for* participants, PPIE needs to be integral within the structures, policies, and processes of the study team so that power is shared [51-54]. This will only occur by building on the existing methods for public engagement using innovative new models encompassing the breadth of patient voices alongside industry, regulators, and academics [55,56].

While “ticking the box” of PPIE is relatively easy, PPIE that is truly representative of the target population and integrated into all phases of the design and research process is much more challenging and therefore costly. However, there are examples of integration of a wider range of patient populations throughout the design, prototype, and evaluation phases (eg, SlowMo and gameChange [see Textbox 4 for details] and mindLAMP [57]) and examples of global applications across LMICs as well as higher-income countries [58]. Textbox 4 provides some more details on 2 examples.

Consideration of the specific characteristics of involved PPIE members is also important. In the move toward an improved user-centered PPIE involvement, researchers should consider involving a broader range of patient experiences. PPIE involvement in product and study development often comes from “expert” PPIE members. While this is helpful (“expert” PPIE members have often had experience with other interventions and can contribute specialist knowledge), input from diverse experiences enhances the breadth of perspectives and understanding throughout the development phases. A risk inherent in participatory design is that the voices of seldom-heard groups are neglected, and so purposive recruitment of PPIE to ensure representation is recommended. This addresses potential hurdles such as digital exclusion (ie, in terms of access and skills) and the monitoring of the negative and positive effects of proposed design solutions for a range of stakeholders [6,41].

**Lived experience perspectives.**

**Overview**

- The inclusion of an individual with lived experience as part of the multidisciplinary panel approach was an important way to ensure that the patient voice was heard and included during the meeting and interwoven throughout the discussions. It was also an opportunity to provide an alternative perspective beyond the parameters of academic research and study.
- The user perspective shared in the meeting itself drew upon a number of key challenges in the current model of mental health care (in the United Kingdom), namely, (1) respect for patient autonomy and voice, (2) sensitivity to cultural barriers, and (3) accessibility of interventions and their adaptability to patients’ lives. While this is the experience of only one individual, it does raise important considerations and can inform 3 key user perspective takeaways from the meeting to guide further work in the study of digital mental health for severe mental illness (SMI).

**Patient autonomy and voice**

- Respect for patient autonomy and listening to the voices of patients are crucially important in the research and development of digital mental health interventions, just as they are in the delivery of care. The lived experience shared during the meeting indicated that there was a feeling of a lack of control and respect for patient decision-making capacity in the provision of care. There was a sentiment expressed that, by stressing the importance of the patient’s voice in the research and production stage of mental health interventions, this may subsequently set the tone for real-world application.
- As discussed in this paper, there is already an identified greater need for genuine coproduction to facilitate this, with coproduction being defined as an equal weight placed on the involvement and accountability of those with lived experience and of academics and experts [48]. A challenge in coproduction continues in current methodologies, language, and ways of working in academia that are not always easily translated or transferred to those outside this field. There needs to be more to ensure that the platform for shared work is an equal one, with opportunities for both parties to learn from one another and ensure that coproduction is suitably accessible for genuine equal input in knowledge production. During the meeting, it was considered from a user perspective that, when principles of coproduction in research are fully realized, this may improve patient uptake and also strengthen patient autonomy. With respect to the latter, this can arise because greater insight into the needs of patients among digital health designers and clinician researchers should be much clearer as a result of patient input as well as shifting standards in mental health care more broadly.

**Cultural barriers**

- Related to the aforementioned, there is a need for understanding regarding cultural barriers and stigma that act as a deterrent to access to mental health interventions and care for different users. There is a plethora of reasons why culture can be a barrier, from traditional roles and responsibilities of an individual that draw on available time, to education on mental illness (or a lack thereof) and particularly negative stigmatization. The user perspective shared during the meeting indicated that many currently available interventions do not offer sufficient nuance or flexibility in recognizing the challenges to access faced by many communities. This is linked to existing literature that often acknowledges the limitation of research being undertaken with a focus on Western-centric, middle-class, digitally literate populations despite high proportions of global mental illness being found in low- and middle-income countries. Both in the early stages of research and development and in the deployment of existing interventions, it is necessary to reflect on which communities have the greatest needs to be heard and understood.

**Accessibility and adaptability**

- For a service user or individual with lived experience of SMI, a best-case scenario is one in which all barriers to accessing an intervention have been removed. An important barrier for many will be flexibility of access. Converging both the points on patient autonomy and overcoming cultural barriers, increased flexibility in access to interventions takes away a number of crucial obstacles. It is here that digital mental health has an opportunity to have a significant impact. Whether owing to the need for discretion, the weight of other time-consuming responsibilities (particularly in the case of high-functioning individuals with SMI, as shared during the discussion), or cultural stigma, the opportunity for adaptability not only serves a practical purpose but also provides a notion of empowerment and a sense of control for the individual.

**Examples of human-centered design in the development and evaluation of digital therapeutics for psychosis.**

**SlowMo: integrating an interventionist causal approach and inclusive human-centered design to develop a next-generation cognitive behavioral therapy for psychosis (CBTp)**

- Background
  - An evidence-based causal reasoning mechanism in paranoia (“fast thinking”) has an antidote in slow thinking (ie, belief flexibility) to improve paranoia and promote living well [59].
  - Proof of concept randomized experiments, and a feasibility randomized controlled trial (RCT) showed CBTp targets fast thinking and promotes slow thinking and improved paranoia [60-63].
- Patient and public involvement and engagement (PPIE) and an inclusive, human-centered design
  - Problem: the need to improve access, experience, and outcomes for the National Institute for Health and Care Excellence (NICE) recommended CBTp, particularly among marginalized groups [64].
  - Method: UK Design Council’s Double Diamond, using ethnographic methods to define the design problem and iteratively co-designing solutions and testing prototypes with purposive sampling of users (n=18). Interdisciplinary collaboration with the Helen Hamlyn Centre for Design, the Royal College of Art, King’s College, London, software developers, and National Health Service (NHS) Trusts.
  - Solution: a redesigned version of the therapy, SlowMo, tailored to users’ needs which supports self-monitoring, provides accessible and memorable information, is enjoyable and trustworthy, promotes personalization, and provides flexible interpersonal support.
    - A web application supports the delivery of sessions with a therapist, which is synchronized with a native mobile app for use in daily life, addressing access and data protection concerns.
    - Responsive, touch screen technology supports personalization and visualization of thoughts and thinking habits.
    - Audiovisual lived experience stories provide engaging interpersonal support.
- Multisite RCT and process evaluation (SlowMo1 [25]; N=361)
  - This was the first digitally supported therapy for paranoia to demonstrate efficacy (improved effect size compared to conventional CBTp was achieved in half of the minimum number of recommended sessions [65]), and the mechanism of change. A process evaluation of therapy experience demonstrated high rates of therapy uptake and adherence [66], a coproduced qualitative interview study supported the user experience and mechanism of change [67], and a user experience study showed that SlowMo bridged the “digital divide” as poorer digital literacy in Black people and older people did not translate to the user experience of SlowMo [19].
  - A PPIE evaluation indicated valued outcomes in the RCT [68].
- Future research includes implementation, effectiveness, and cost-effectiveness study (SlowMo2; ongoing)
  - Interdisciplinary co-design of software for implementation incorporating lived experience findings from the RCT.
  - Lived experience coapplicant and leadership.
  - Substantive posts for lived experienced researchers and purposive recruitment of a representative lived experience advisory panel.
  - The NICE early value assessment has recommended SlowMo for use in the NHS for the treatment of paranoia in adults with psychosis while more evidence is generated [69].

**gameChange: automated virtual reality (VR) cognitive therapy for treating agoraphobic avoidance and distress in patients with psychosis**

- Target
  - Agoraphobic avoidance in psychosis
- Defining the clinical problem
  - A survey of 1809 people with psychosis [70] showed two-thirds had levels of anxious avoidance comparable to agoraphobia.
- Steps of user involvement
  - Aimed to create a VR therapy to help people with psychosis feel safer, more confident, and in control in everyday situations.
  - The design brief: 3 hours of novel VR experiences with graded levels of difficulty. Automated through a virtual coach, users would be guided through 6 scenarios and given opportunities to drop their defenses and test their fear beliefs.
  - A person-centered design approach was used involving people with psychosis at each stage of development, including choosing scenarios, selecting tasks, testing prototypes, and user testing [71].
  - In total, >100 people with psychosis provided ≥500 hours of input.
  - gameChange is Conformité Européenne (CE) marked as a Class I active medical device.
- Clinical testing and implementation
  - Clinical testing in a multisite RCT [72].
  - Health economics evaluation was embedded in the clinical trial [73].
  - gameChange is approved for use in the NHS while more evidence is generated to treat severe agoraphobic avoidance in people with psychosis aged ≥16 years (with the support of a mental health professional) [74].

##### Personalized Approaches

Facilitating personalization of digital approaches increases their ability to meet the diverse needs of people in real-world clinical populations [10]. Incorporating patient preferences in the broad development of tools is essential, but integrating patient preferences into the operation of the digital tool also enables genuine personalization so that the tool is closely matched to the individual requirements and preferences of the user [75]. A concrete step toward personalized approaches can include increasing the pool of data that informs the use of an intervention. One route to this increased body of data would be to facilitate data sharing from all studies in a specific area (see also the *Theme 2: Research Methodology* section). While there are specific ethical challenges in data sharing, particularly in digital mental health studies where sensitive personal data are often collected, there are well-defined principles to guide best practice [76]. Despite this, digital health data sharing remains less common, although there is a clear recognition of the need for this [76].

##### Early Interventions

Early intervention for psychosis has the potential to identify and improve the outcome for individuals who meet clinical high-risk state for psychosis (CHR-P) or first-episode psychosis (FEP) criteria, but most individuals with CHR-P who later develop psychosis are not currently detected during the prodromal phase [77]. Most individuals with FEP use web-based resources, and 76% responded favorably to the possibility of receiving web-based mental health support [78] and so could be identified through web-based approaches [79]. However, such tools need to be appropriate and appealing for this age group—social media and low-threshold entry points may be useful to extend the early intervention approach outside established care pathways [80]. In addition, evidence-based digital tools and resources are needed to guide parents, carers, and the young person’s wider support networks (such as teachers or social workers). Young people also need to be actively involved in coproduction to ensure the highest-quality equitable outcomes [81].

### Theme 2: Research Methodology

#### Current Challenges

A key challenge for all areas of digital mental health is the lack of validated and internationally agreed standards for digital biomarkers or objective behavioral data obtained from patients’ personal devices [6,82]. This means that studies vary in how they assess digital biomarkers and interventions, and in what and how they measure change, engagement, adherence, improvement, or potential harms. For instance, with some exceptions, studies have not examined adverse events [38]. This is needed for safety measurements in digital interventions not only among people with SMI but also in health care more broadly. In 2023, regulatory bodies such as the US Food and Drug Administration (FDA) issued draft guidance outlining how verification and validation of digital health technologies should be approached [83], including some challenges that are more prominent in digital health, such as data privacy and confidentiality. However, a 2023 review of FDA approvals across all domains of health care, including digital health technologies, suggested a lack of scientific rigor across studies and the need for higher-quality research [84]. Because there is significant variability between studies, current research is not easy to replicate or validate, leading to reduced confidence in the results and the robustness of the evidence base supporting digital interventions. In addition, digital mental health research in recent years has focused mainly on reactive interventions that address immediate or short-term needs, whereas longer-term and preventative approaches or time series–aware methods often remain lacking [85].

#### Potential Solutions

##### Agreed Standards for Digital Mental Health Research

Differences regarding study standards and data quality need to be addressed at an international level with agreed standards for studies and their subsequent publication. The homogenization of standards could mitigate fragmented approaches, and regulatory agencies and funders could insist on such standards [86]. This could prompt researchers to align research protocols in digital mental health so that there is transparent reporting of the data collected and the frequency with which they are collected (which would allow for comparison and combination of data sets). This would also facilitate the understanding of the relative usefulness of, for example, particular machine learning paradigms (as these are most accurate on data sets that are similar to those they have been trained on), and this would require separate data processing standards [87,88].

##### Data Sharing

To enable the comparison and combination of data sets, standards should require the sharing of data via open-source documentation (eg, the study by Bent et al [89]). While this can generate some potential ethical challenges (Textbox 5), there are already international examples demonstrating data sharing [90,91]. The older 2016 CrossCheck study of people with schizophrenia using a smartphone app to collect digital phenotyping data also offers an open data set that has enabled numerous publications advancing computational methods of symptom and relapse prediction [92].

**Applying a biomedical ethics framework in the use of digital tools for severe mental illness (SMI).**

**Respect for autonomy**

- Digital phenotyping involves continuous, real-time multimodal streaming of data from smartphones and other internet-enabled devices. As noted, the insights offered by these data may be highly sensitive, uncovering, for example, an internet browsing history, purchasing patterns, or levels of social contact. Conceivably, such data may also create legal exposures. For example, an estimated 1 in 4 people with SMI also experience substance use disorders [93], which, in some circumstances, could heighten risks of criminal behavior or illicit drug purchasing or use. For clinicians to access these digital data streams to yield benefits for patients, health care users must provide fully informed consent regarding the whys, hows, and consequences of sharing digital information, including which data may yield the most valuable insights and how health care providers will store and use this information.

**Beneficence**

- Digital tools have the potential to offer faster, less expensive, and more accessible care at scale. Many digital interventions, such as apps, also afford patients with SMI opportunities to engage with technology without the fear or risks of stigmatization that may arise in clinic visits. While some tools already offer considerable promise, for the benefits of these applications to be optimized, greater research is needed to explore implementation to increase uptake (themes 1, 2, and 4).

**Harms**

- Clinicians are duty bound to “first, do no harm,” yet, currently, there is a deficit of research into the potential adverse effects of digital health tools for SMI (theme 2). Identifying when tools lead to incidents of harm, including self-harm or discontinuation of treatment is critical to ensure safety. For example, preoccupation with checking health-tracking data via downloadable apps and wearable devices might increase anxiety, especially among some subpopulations of patients with SMI.

**Justice**

- Artificial intelligence–powered digital tools rely on patient involvement, and if data sets are not representative of the populations in which they will be used, these technologies may not be as useful or could be harmful for these groups. Furthermore, to benefit all patients, digital tools need to be accessible to everyone [94]. While digital divides are narrowing, the most vulnerable patients—including those with SMI—are often more likely to live with lower incomes, meaning that they are still less likely to own digital devices, to have access to broadband, or have acquired the digital literacy skills necessary to partake in technology use and reap the benefits [95]. In many countries, advancing digital health research is now a priority [96]; however, without concerted efforts to improve the distribution and access to these tools, inequities will persist or potentially increase. Aimed at improving digital literacy among patients, including those with SMI, the Digital Opportunities for Outcomes in Recovery Services program has been deployed in many community settings, including in the United Kingdom and the United States [20].

**Professional-patient relationships**

- Used effectively, digital tools are unlikely to replace human relationships, including in care settings. Moreover, for data to be effectively interpreted and understood, a deeper and more honest and trusting partnership between patients and clinicians is imperative. For example, the insights gleaned from digital phenotyping are fallible and require context—only patients with lived experience can assist in offering the situational knowledge needed to furnish a deeper understanding of what the data show and how they might be harnessed in preventative care [94]. Conversely, as noted previously, owing to the nature of data gathering, there are also multiple new opportunities for these technologies to undermine trust in the fiduciary clinician-patient relationship (see the previous points). Regulatory policies, civic debate and patient involvement, and health professional ethical awareness must strive to keep abreast of advances (themes 1 and 3).

##### Measuring Harms

For transparency, standards should also require that potential adverse events or harms, as well as benefits, are identified and measured (eg, the International Collaboration for Harmonising Adverse Events Reporting in Technology for Schizophrenia (iCHARTS) network by Bucci et al [97]). In addition, preregistration of protocols [98] should also be implemented to increase the publication of “negative” findings. This would balance the known bias toward the publication of “positive results”—a trend already noted in biomarker research for bipolar disorder [99]. Such approaches could better inform researchers about when to build interventions using existing platforms and when novel platforms are needed. Harm need not be limited to classic symptom exacerbation and can also include loss of privacy, inequality or discrimination, social or personal loss, and even physical injury. These could be measured using a combination of both self-report and investigator-assessed scales.

##### Prevention and Longer-Term Strategies

Digital mental health research in recent years has focused mainly on reactive interventions in the short term, but longer-term or preventative interventions may be equally or more important, particularly in areas such as SMI and suicide prevention. Examples include the use of web-based platforms in suicide prevention while tackling harmful content that could promote or encourage suicide and self-harm [100-103], and web-based screening in youth mental health to support the identification of high-risk individuals or groups.

##### Mechanistic Research in Digital Mental Health

Research on the mechanisms of digital mental health is needed, specifically more focused research on the potential moderators or mediators of effects. Even engagement itself may require mechanistic research as it has proven to be a challenging construct to improve upon. For example, a recent review of digital therapeutics suggested that the field could benefit from the application of clinical pharmacology principles from the drug development field, such as a stepwise and progressive focus on engagement and adherence, proxy of effects, and clinical end points [104]. This would need funders to launch specific calls for this type of research (as this is intricate and costly) and is explored further in theme 3 in the following section.

### Theme 3: Regulation, Governance, and Funding

#### Current Challenges

##### Funding

Funding is a major issue specifically in digital mental health as the process is often fragmented [105] and may only cover the initial development of digital interventions. The rest of the pathway is also often lengthy and costly, but is critical in the road toward implementation and needs to incorporate the processes of regulation and market transfer. A particularly important area to assess is the implementation of a mental health digital product in the real-world health care setting. This will involve a comprehensive analysis of implementation costs, integration into the existing IT infrastructures of the institution, and regulatory and privacy compliance, but this may not have been adequately assessed at the beginning of the process. A result is that many digital health technologies are not iterated and sustained, with a 2022 review finding that nearly half of apps created for schizophrenia research in the last decade are no longer accessible or supported [11]. These challenges in funding impede replication and impair the research-to-clinical translation of digital mental health tools (see further discussion in theme 4). An area of additional challenge is that research funding has usually not considered the complexity of science (including the need for significant user input throughout the research cycle, as explored in theme 1). In addition, digital interventions have technological complexities that have a significant impact on costs, such as the need to maintain apps on an ongoing basis, provide updates, and implement new and regular cybersecurity and data protection measures.

##### Regulation

These challenges are complicated by a lack of health care regulation specifically tailored for the digital space. For example, in the United States, many digital interventions fall within FDA regulations that are challenging when applied to this area, and so many interventions do not come to market. There are also other extra regulatory issues that need to be formally addressed regarding safety and data protection, including privacy and security [106]. For example, data collection in digital phenotyping involves tracking patients beyond traditional health information and can involve data such as social media posts, geolocation, and telephone and SMS text message traffic (among other data) that can provide revealing insights about the daily lives of individuals [107,108]. Studies also show that there are significant limitations with clinicians’ awareness of the ethical considerations regarding artificial intelligence–powered innovations in health care [109-113] (Textbox 5). Furthermore, health laws have not kept up with digital technologies, although authorities have recently made efforts to regulate technology while also aiming to protect civil liberties and rights to privacy [107,108].

#### Potential Solutions

Solutions include advocating for governance change, but this would need to ensure the involvement of and contributions from multiple stakeholders (patients and carers, technology experts, researchers, clinicians, health technology assessment agencies, and regulators). One option as a model might be a roundtable discussion with all relevant stakeholders, such as that at the recent UK AI Safety Summit [114,115]. Governance change could also encompass standards for the level of evidence required, including controlling for digital placebo effects and demonstrating savings in cost [116]. In line with governance change, funding in this area needs to be more adaptive to recognize the particular needs in this space (eg, commitments to funding until completion or funding the people rather than the project [117]). As an intermediate step, guidelines for best practices and research standards that funders and journals may enforce would also shift the field in a positive direction. In terms of ethical concerns, including how to navigate evolving regulations, clinicians will require greater training, and patients will need more guidance and advice on the benefits and potential risks of these digital tools in health care settings (Textbox 5). Brief training interventions should be considered for all stakeholders. For example, among patients, the use of “digital navigators”—peer supporters who can offer patients advice on how to download and use apps, where to find information about privacy considerations, and the evidence base for these tools—has been pioneered in outpatient psychiatry with notable success [118].

### Theme 4: Implementation in Real-World Clinical Settings

#### Current Challenges

Translation of research findings into the clinical setting is a significant challenge. In general, many digital health studies fail to reach the market or translate into real-world clinical care, and even when they do, rates of adoption can be low [119]. For example, in a study of smartphone apps for schizophrenia, <10 of those identified from a search of interventions from the research literature were easily accessible to the population, and the picture was similar in a parallel search of marketplace apps [11]. These were also few in number and lacked frequent updates (average time since last update 1121 days). Even where there is engagement in the research setting, sustaining this in the clinic, outside of the constraints and incentives of clinical trials, is challenging, particularly over the longer term. For example, digital health tools with 44% to 99% completion rates in research studies translate to only 1% to 28% in actual clinical use [120].

There are several potential reasons for this:

1. Researchers are often not trained in the technology skills and application of the implementation science methods required to ensure the tailoring and ongoing development of interventions for real-world uptake.
2. Even once digital interventions have been fully developed, individual clinicians may be reluctant to implement them in the clinic. Uptake requires behavior change from clinicians, who may be resistant due to lack of training, skills, or confidence in digital interventions, regulatory issues, and cautions and anxieties about adopting commercial therapy products.
3. Patients, carers, and the public may also be reluctant due to digital inclusion issues such as digital literacy and confidence (especially for some groups, such as older patients or patients who are severely ill [121]) as well as concerns about privacy and digital coercion [122].

In addition, the focus of digital mental health research may contribute to difficulties in translation into real-world settings:

1. Many research studies focus on “replacing” the clinician, with apps designed for stand-alone use, whereas the research base suggests that blended or augmented care, in line with patient preference, is more beneficial [16,118,123].
2. Many studies focus on general outcomes rather than the mechanisms of change, and without registration of studies, there is a risk of inefficient use of funding resources.
3. The focus of digital mental health research also tends to be on biological and psychological parameters, whereas social mechanisms are equally important [10].
4. For the user, there is a lack of guidance on which interventions might be better or worse. This is a long-standing problem [124] and reflects the lack of evidence base. For example, assessments of smartphone tools for suicide prevention have identified a wide variety of approaches but also include apps with potentially harmful content [125,126].

#### Potential Solutions

Successful development, implementation, and application in real-world clinical settings will require collaboration between multidisciplinary groups who have not traditionally been brought together, for example, a wider range of academics with complementary expertise, clinicians, user groups and industrial designers, software developers, commercial partners, and regulatory specialists. Skills and confidence in using digital interventions will be a key element. Training schedules for clinicians in using and integrating digital tools into their clinical practice have already been defined, and these need to be integrated into clinical training at all levels from core training to specialist academic programs [127], with health care organizations creating sustained budgets to fund digital inclusion schemes. Patient and user confidence is also important, and there are examples of effective training schemes for SMI (eg, the study by Hoffman et al [20]) and of interventions to help bridge the technological divide, such as digital navigators [118] and specialized youth mental health workers [44].

Workable and scalable solutions will rest in augmenting the in-person consultation using a blended approach. In addition, the focus needs to be on increasing the capacity of clinical services, such as by augmenting service provision with support workers who can deliver protocolized interventions. Implementation science frameworks can be used to guide the assessment of site readiness and evaluate their ability to successfully introduce, implement, and sustain digital technology use [119,128].

Solutions will also depend on looking at social interventions, which may be population focused as well as targeted at the individual or specific group level. Examples include wide-reaching digital training, awareness and antistigma campaigns [129], and automated solutions to reduce access and exposure to lethal means of suicide [130-132]. Although in the past there have been difficulties in formal guidance for the user [121], the American Psychiatric Association app evaluation framework offers a viable alternative [133].

## Discussion

### Overview

In this paper, we have illustrated and discussed the complexities of collecting data, delivering treatment, and the ethical challenges of digital mental health in the care pathways of people with SMI. During the process of the consensus meeting and the consensus recommendations, we implemented a thematic approach focusing broadly on digital interventions for psychosis. This enabled us to concentrate on an area of significant need where digital health innovation has the potential to be safe and effective [134]. However, we also found that, in taking this thematic approach, we identified broader issues, and the solutions proposed can apply to other fields of mental health.

### Potential Limitations

There are some potential limitations to our approach. While we conducted a systematic review of the literature, we restricted this to one source (PubMed) and to articles published in the last 5 years on psychosis and schizophrenia. The primary aim of this review was to ensure that the panel was informed before the meeting, but it is possible that this approach may have missed some relevant publications (eg, on other diagnoses within SMI, such as bipolar disorder). In addition, as with all consensus meetings, there are no standard guidelines for identifying expertise. Although we selected participants to represent a diverse spectrum of views, the reliability of consensus opinions is dependent on the specialist knowledge and experiences of those who participated. We aimed to encompass a wide variety of expertise in the expert group and panel to ensure that many different perspectives were heard. These included clinical psychology, psychotherapy, psychiatry, philosophy of medicine and health care ethics, health services research, social sciences, health informatics, digital health care, and mental health charity management. We included an expert with lived experience of SMI in the panel but recognize that we could have included more, which we take on board for future consensus studies. The structured in-person nature of the meeting may also have unintentionally excluded the opinions of experts, particularly those with lived experience of SMI, who are not willing or able to engage in that format. However, we had 2 PPI contributors within the process (one in the panel and one in the expert group) who made material contributions throughout; to the literature review, presentation and discussion of the evidence, formation of consensus, and coproduction of the paper. In this way, we aimed to engage a high-quality PPI coproduction rather than focusing purely on the number of PPI members involved. In addition, by adopting and building on new features of the consensus method used in our previous work [6] (eg, including a panel separate from the expert group and including PPIE members in the expert group and panel), we have strengthened international and multidisciplinary discussion on this important but often overlooked area [134].

### Principal Findings

The consensus meeting identified a number of broad recommendations in this field:

1. A new approach to research in digital mental health is needed that is different from the standard pathways used in pharmacological and psychological intervention research.
2. This new approach requires internationally agreed standards for reporting research and open data access to allow for true collaboration and enable easy validation of biomarkers and replication of interventions.
3. This could be facilitated by the development of shared protocols for research to be carried out in multiple recruiting sites.
4. Research should place an equal emphasis on social and population factors [135] as well as biological and social factors in the etiology and maintenance of symptoms and risk in SMI.
5. Successful implementation and application of digital mental health in real-world clinical settings will require new and evolving collaborations between academics, clinicians, people with lived experience of SMI, industrial designers, software developers, and regulatory specialists.
6. The uniqueness of the digital space in clinical research means that it requires a different approach, focusing not just on the translational pathway of research in isolation but also on the business model; the “product”; and the impact or value of the intervention for all relevant stakeholders, from patients to clinicians, health care organizations, and society at large.
7. This new approach may prompt a possible conflict of values as the “product” (the digital mental health intervention) needs to be economically viable so that it can be scaled and sustained while also ensuring effectiveness, safety, and compliance with medical technology quality standards and health care regulation.
8. Potential harms are just as important to record as in other areas of research (such as with pharmacological or psychological interventions) but may be more hidden and need to be actively sought out and logged.
9. Funding structures need to be adjusted to the new elements required within digital mental health research and must be sufficient to support ongoing product development in line with regulatory requirements and allow for representative PPIE.
10. Funding streams will need to recognize that not all the tasks required can be performed by a single person or group and they may need a “relay” approach between stakeholders (ie, projects led by clinician academics at the proof-of-concept, feasibility, and efficacy stages, with increased commercialization and regulatory input as products move into implementation and market).
11. In addition, more fundamental organizational changes are needed to underpin necessary changes to funding and research study approaches. Participants with lived experience and academic experts do not always “speak the same language” [136], so awareness of differences in expression and the need to work together to solve health problems is necessary to minimize the impact of power imbalances and promote coproduction [137]. This can be achieved by developing safe spaces to create and share knowledge [138] and allow for opportunities for researchers and participants with lived experience to learn and enrich their own expertise from the experience of informed participation and collaboration [49]. This improved communication could provide the platform to create new models of care to deliver digital services, which will also require adjustments to organizational structure, policies, and membership.
12. The ethical components of digital mental health are also crucial. This is not just in terms of trust and trustworthiness in digital mental health but also in managing patient expectations, “ownership of their own health,” and future developments.

### Conclusions

In this study, our approach, which combined an international expert meeting with PPIE throughout the process, consensus methodology, discussion, and publication, was a fruitful way to reach expert consensus and focus directions for future research and clinical implementation, especially in rapidly evolving fields. We improved and expanded our approach and showed how to integrate research evidence with a process of measuring real-world clinical impact over time [139]. To enhance our scope, future meetings should directly involve stakeholders in health technology assessment and representatives from regulatory agencies and industry and encompass researchers working in health care ethics and policy in different countries, including from the Global South. Similar initiatives should be repeated regularly in digital mental health and adopted also by researchers in other fields to focus research and organizational change to effect real-world clinical implementation.

## Abbreviations

CDC: consensus development conference
CHR-P: clinical high-risk state for psychosis
FDA: Food and Drug Administration
FEP: first-episode psychosis
GALENOS: Global Alliance for Living Evidence on Anxiety, Depression, and Psychosis
LMIC: low- and middle-income country
PPI: patient and public involvement
PPIE: patient and public involvement and engagement
SMI: severe mental illness

## Data Availability

All data generated or analyzed during this study are included in this published article.
